# Structures of SHV-1 β-Lactamase with Penem and Penam Sulfone Inhibitors That Form Cyclic Intermediates Stabilized by Carbonyl Conjugation

**DOI:** 10.1371/journal.pone.0049035

**Published:** 2012-11-08

**Authors:** Wei Ke, Priyaranjan Pattanaik, Christopher R. Bethel, Anjaneyulu Sheri, John D. Buynak, Robert A. Bonomo, Focco van den Akker

**Affiliations:** 1 Department of Biochemistry, Case Western Reserve University, Cleveland, Ohio, United States of America; 2 Department of Medicine, Pharmacology, and Molecular Biology and Microbiology, Case Western Reserve University, Cleveland, Ohio, United States of America; 3 Research Service, Louis Stokes Cleveland Department of Veterans Affairs Medical Center, Cleveland, Ohio, United States of America; 4 Department of Chemistry, Southern Methodist University, Dallas, Texas, United States of America; Monash University, Australia

## Abstract

Bacterial β-lactamase enzymes are in large part responsible for the decreased ability of β-lactam antibiotics to combat infections. The inability to overcome β-lactamase mediated resistance spurred the development of inhibitors with penems and penam sulfones being amongst the most potent and broad spectrum mechanism-based inactivators. These inhibitors form covalent, “suicide-type” inhibitory intermediates that are attached to the catalytic S70 residue. To further probe the details of the mechanism of β-lactamase inhibition by these novel compounds, we determined the crystal structures of SHV-1 bound with penem **1**, and penam sulfones SA1-204 and SA3-53. Comparison with each other and with previously determined crystal structures of members of these classes of inhibitors suggests that the final conformation of the covalent adduct can vary greatly amongst the complex structures. In contrast, a common theme of carbonyl conjugation as a mechanism to avoid deacylation emerges despite that the penem and penam sulfone inhibitors form different types of intermediates. The detailed insights gained from this study could be used to further improve new mechanism-based inhibitors of these common class A serine β-lactamases.

## Introduction

Bacterial β-lactamases in Gram negative bacteria are primarily responsible for the inactivation of our current β-lactam antibiotics. The continued introduction of newer β-lactam antibiotics and β-lactamase inhibitors to overcome β-lactam resistance has been driven by the increased number of β-lactamases including extended-spectrum (ESBL), carbapenem hydrolyzing, and inhibitor-resistant phenotypes (IR) [1]. In addition to the three clinically used β-lactamase inhibitors (clavulanic acid, sulbactam, and tazobactam) a number of other mechanism-based inactivators are being explored that employ a variety of different chemical pathways to achieve inhibition [2]. One potentially advantageous strategy is to develop “suicide-type” inhibitors that undergo additional chemistry once covalently bound to the enzyme. This chemistry render the inhibitors less susceptible to deacylation. Here, the underlying chemical rationale is to form a stable acyl enzyme or “long-lived intermediate” that hinders the hydrolytic activity of the β-lactamase while the partner β-lactam traverses the periplasmic space and inhibits the cell wall transpeptidases.

**Figure 1:**
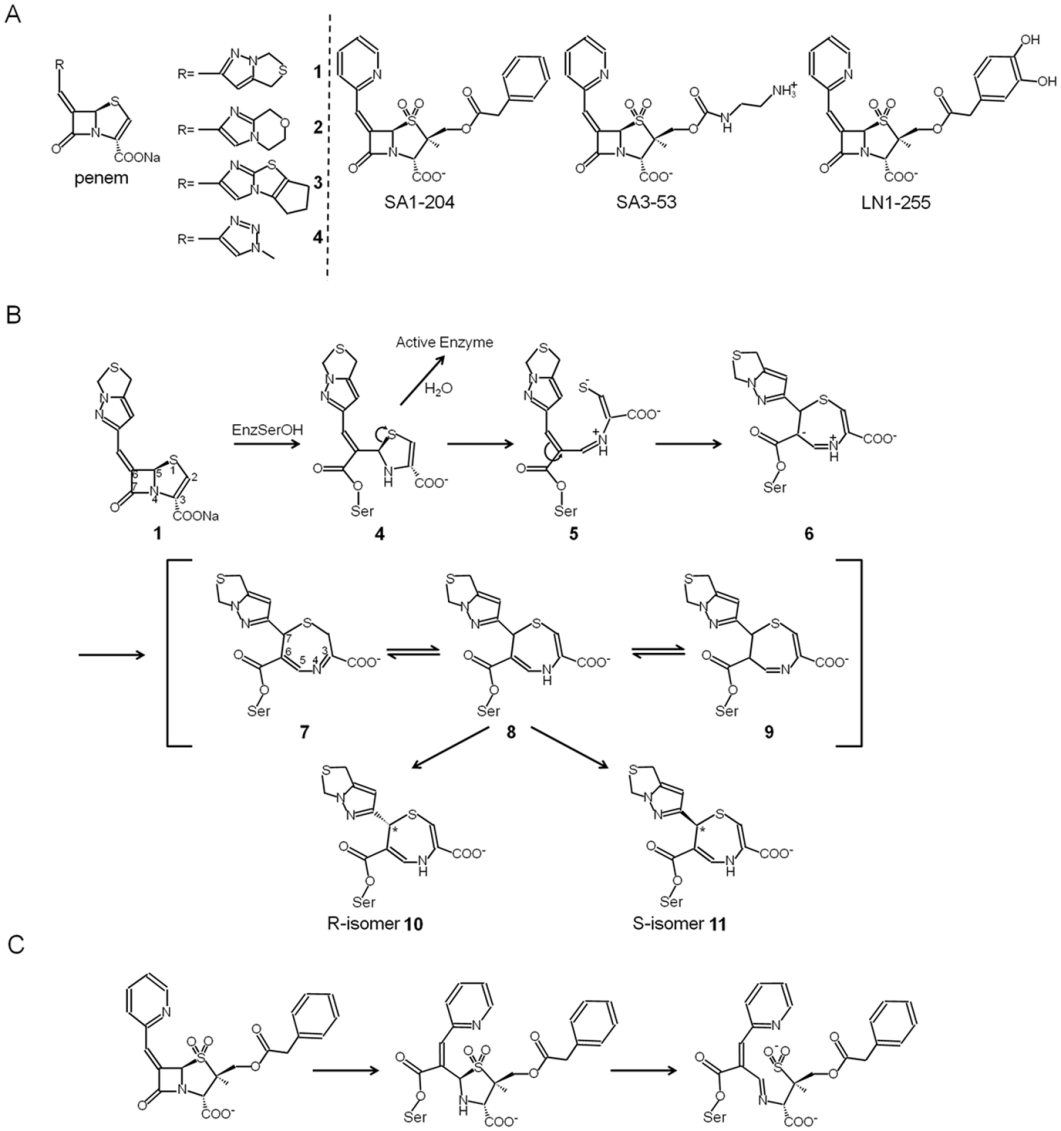
Penem and penam sulfones and their reaction mechanisms. (A) Chemical structures of penem and penam sulfone compounds. (B) proposed inhibition mechanism by a penem **1** (based on Knox’s work and others) [10], [11]; carbon atoms labeled with * are the stereo centers; (C) proposed reaction mechanism of SA1-204.

Penem and penam sulfone β-lactamase inhibitors bearing heterocycle substitutions at the C6 position via a methylidene linkage (see Figure 1) are two compound classes that inactivate class A β-lactamases by forming a long-lived intermediates [2]. Despite their similarities, these penem and penam inhibitors undergo different cyclization reactions forming distinct long-lived cyclic inhibitory intermediates. Penem and penam sulfones have broad inhibitory potency against Class A, C, and D β-lactamases with nanomolar IC50 values [3]–[6] and some even have activity against Class B metallo-β-lactamases [7]. As a result of their potency and ability to inhibit many different β-lactamases, selected representative compounds of the penam and penem classes have been studied in depth using mass spectrometry and protein crystallography to probe their binding mode to different β-lactamases [5], [8]–[11].

Intriguing hypotheses regarding class A β-lactamases and penems and penam sulfones have been put forth. For example, the relatively unusual *S* enantiomer of the 1,4-dihydrothiazepine intermediate in class A β-lactamases was predicted [6] and inhibition by SA1-204 was thought to occur via Michaelis-Menten complexes [12]. To further understand the steps involved in the mechanism of inhibition by these compounds, we selected penem **1** and **2** penam sulfones, (SA1-204, and SA3-53) to examine their mode of inhibition against a Class A β-lactamase, SHV-1. Penem **1** [3], [6], [13], [14] and SA1-204/SA3-53 [4], [7], [8], [12] are amongst the most potent inhibitors from the penem and penam sulfone inhibitor classes, respectively. The compounds first form a covalent bond with catalytic S70 concomitant with opening of the β-lactam ring, thus forming an acyl enzyme, followed by opening of the second ring. Penems subsequently undergo 7-*endo trig* rearrangement (cyclization) reaction leading to a 1,4-dihydrothiazepine acyl-enzyme complex (Figure 1B). In contrast, penam sulfones undergo a pyridine-mediated cyclization forming a bicyclic stable intermediate (Figure 1C).

The crystal structures presented here allow us to explain differences and similarities in their inhibition mechanism with each other and compare those to previously determined related complex structures. In addition, these studies offer insights into how different substituents at the C2 and C6 position affect the mechanism of inhibition of class A β-lactamases regarding both the type of stereochemical enantiomer being formed, such as for penem **1**, as well as the final conformation of the stable cyclized intermediate. Deacylation is apparently a slow enough process to allow for such cyclization to occur and the substituents and active site characteristics have a likely significant influence on this inhibitory reaction pathway.

**Figure 2:**
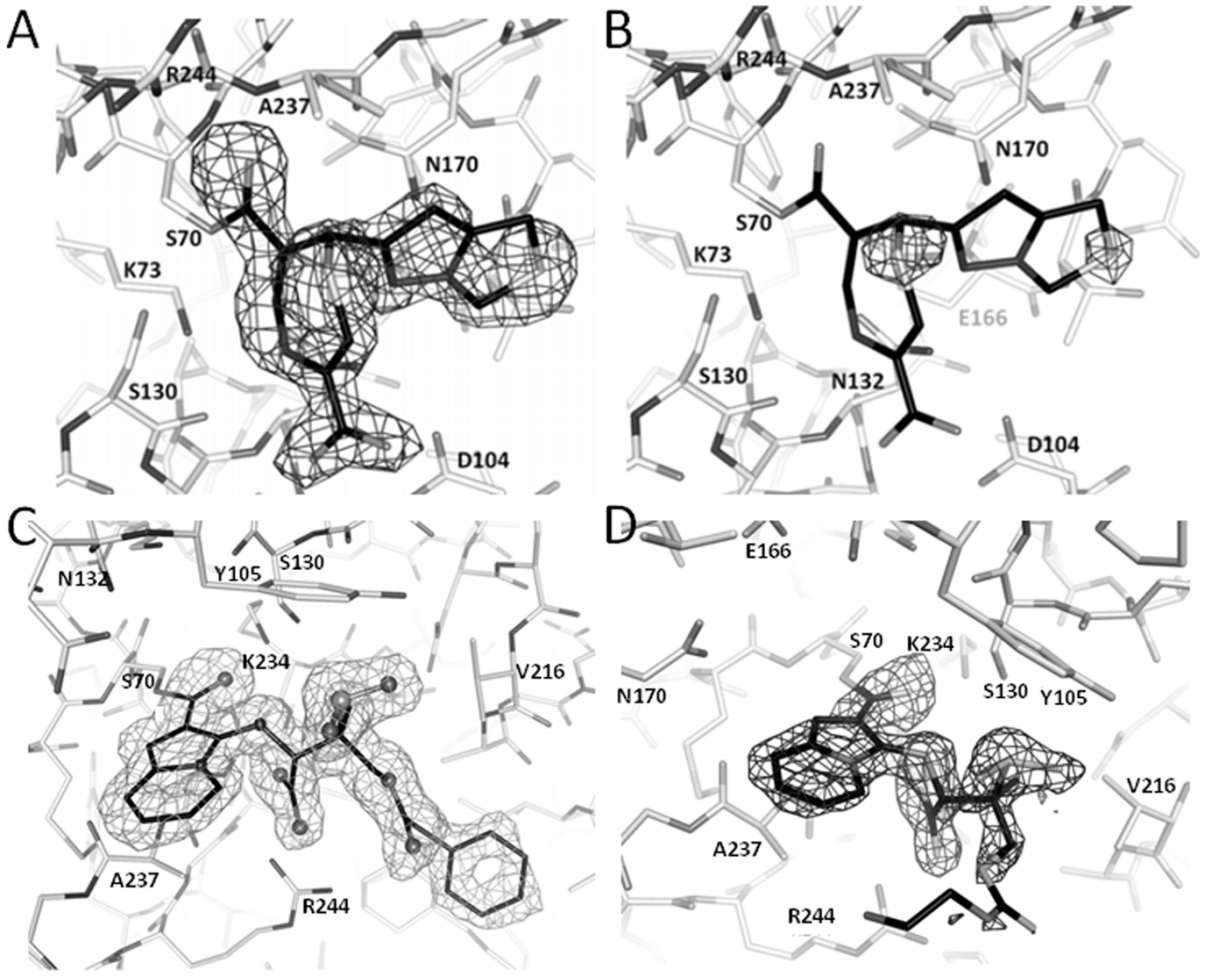
Electron density maps showing inhibitor density in SHV-1 active site (A) Left figure is the unbiased omit *F*o-*F*c map contoured at 3.0σ of SHV-1: penem 1 complex (B) anomalous difference Fourier map contoured at 3.5σ showing strong density peaks on top of the two sulfur atoms of penem 1 intermediate; (C) Unbiased omit *F*o-*F*c map contoured at 2.5σ of SHV-1:SA1-204 complex. (D) Unbiased omit *F*o-*F*c map contoured at 3σ of SHV-1:SA3-53 complex.

**Table 1:** Data collection and refinement statistics.

	SHV-1 : penem 1	SHV-1 : SA1-204	SHV-1 : SA3-53
*PDB identifier*	4GDB	4GD6	4GD8
***Data collection***			
Space group	P2_1_2_1_2_1_	P2_1_2_1_2_1_	P2_1_2_1_2_1_
Cell dimensions			
a, b, c (Å)	49.58, 55.50, 85.80	49.64, 55.58, 83.35	49.39, 55.46, 83.65
α, β, γ (deg)	90, 90, 90	90, 90, 90	90, 90, 90
wavelength (Å)	1.54178	1.0810	0.979
resolution (Å)^a^	50.00−1.84 (1.91−1.84)	50.00−1.53 (1.58−1.53)	46.22−1.60 (1.66−1.60)
R_sym_	7.2 (17.3)	9.2 (29.1)	7.0 (28.0)
I/σI	26.6 (8.1)	39.4 (3.8)	8.3 (2.5)
Completeness (%)	98.2 (90.7)	97.8 (84.9)	99.6 (99.5)
Redundancy	11.9 (9.8)	6.3 (4.7)	3.5 (3.3)
***Refinement***			
Resolution range (Å)	20.01−1.84 (1.89−1.84)	27.68−1.53 (1.57−1.53)	36.89−1.60 (1.64−1.60)
no. of reflections	19909	32972	29342
R_work_/R_free_	18.7/23.4 (25.4/37.1)	16.8/19.4 (23.4/31.6)	19.8/24.2 (33.0/40.0)
r.m.s.d.^b^			
bond length (Å)	0.006	0.009	0.017
bond angles (deg)	1.272	1.306	1.741
average B-factors (Å^2^)			
protein, inhib., wat., others	14.4, 18.9, 29.9, 26.5	16.3, 19.0, 31.1, 26.3	15.4, 32.2, 24.7, 25.1
Ramachandran plot statisctics (%)			
core regions	93.5	90.9	92.2
additional allowed reg.	6.5	8.7	6.9
generously allowed reg.	0.0	0.4	0.9
disallowed regions	0.0	0.0	0.0

a Numbers in parentheses refer to the highest resolution shell.

b rmsd, root-mean-square deviation.

## Materials and Methods

### Enzyme Purification

SHV-1 β-lactamase was expressed and purified as described previously [15], [16]. Briefly, the SHV-1 β-lactamase gene was subcloned into pBC SK (−) vector (Stratagene) and transformed into *Escherichia coli* DH10B cells (Invitrogen). The cells were grown overnight in lysogeny broth (LB) supplemented with 20 µg/ml chloramphenicol to express the protein. After cell lysis via stringent periplasmic fractionation, SHV-1 was purified to homogeneity by two steps using preparative isoelectric focusing and Superdex-75 gel filtration FPLC. Protein purity was assessed using SDS-PAGE; the purified protein was concentrated to 5 mg/ml using a 10 K MWCO centrifugal concentrator (Amicon).

**Figure 3:**
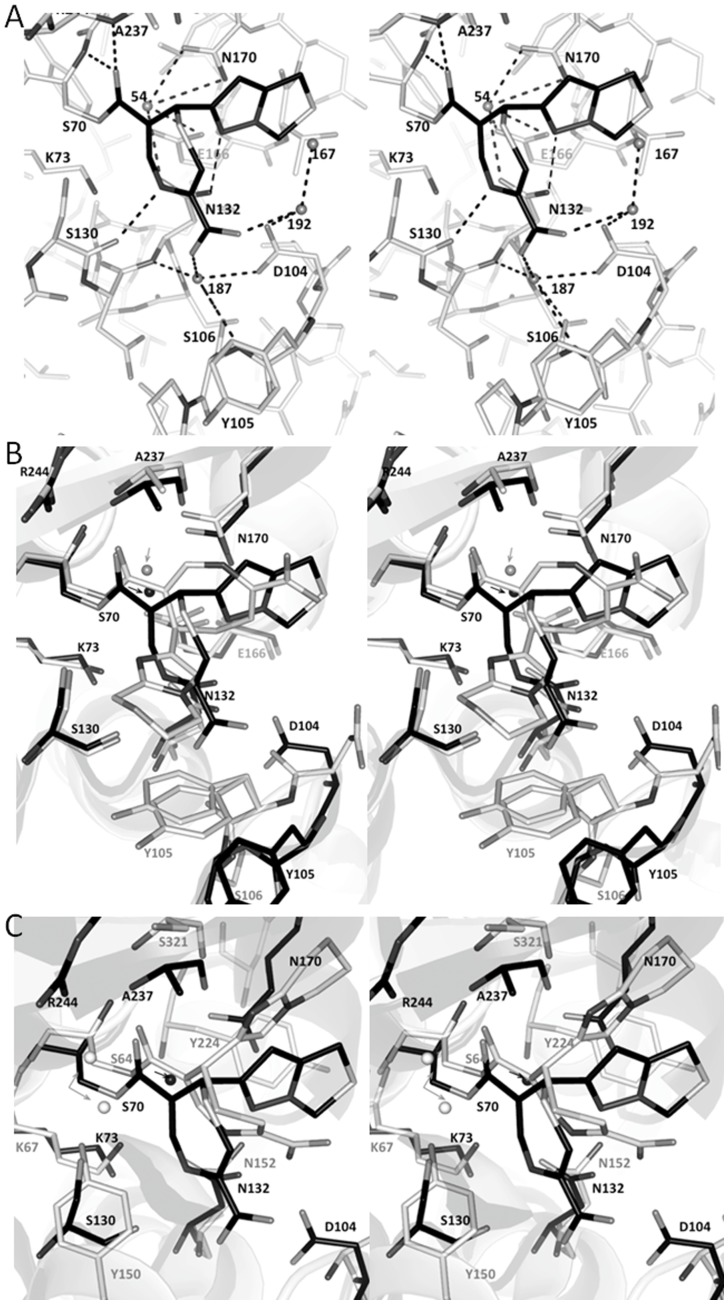
Penem 1 in SHV-1 active site. ( A) Stereo view of penem **1** interactions in SHV-1 active site. Hydrogen bonds are shown as dashed lines. (B) Stereo view of superpositioning of penem **1**:SHV-1 (black) and penem **2**:SHV-1 structures (grey). (C) Stereo view of superpositioning of penem **1**:SHV-1 (black) and penem **2**:GC1 β-lactamase (grey).

### Crystallization and Soaking

SHV-1 β-lactamase was crystallized as described previously [15], [16]. A 5 µl drop containing 2 mg/ml SHV-1 β-lactamase and 0.56 mM Cymal-6 (Hampton Research) in reservoir solution (20–30% PEG 6000 in 100 mM HEPES pH7.0) was equilibrated against 1 ml reservoir solution. Crystals grew in 2–3 days. To obtain the SHV-1: penem **1** complex, crystals were soaked in mother liquor containing 40 mM penem **1** for 21 hours. For the SHV-1:SA1-204 and SHV-1:SA3-53 complexes, crystals were soaked in mother liquor containing 50 mM of their respective inhibitor for 90 minutes and 24 hrs, respectively. After soaking, the crystals were cryo-protected with 20–25% 2-methyl-2,4-pentanediol (MPD) in mother liquor containing the corresponding inhibitor and flash frozen in liquid nitrogen prior to data collection.

**Figure 4:**
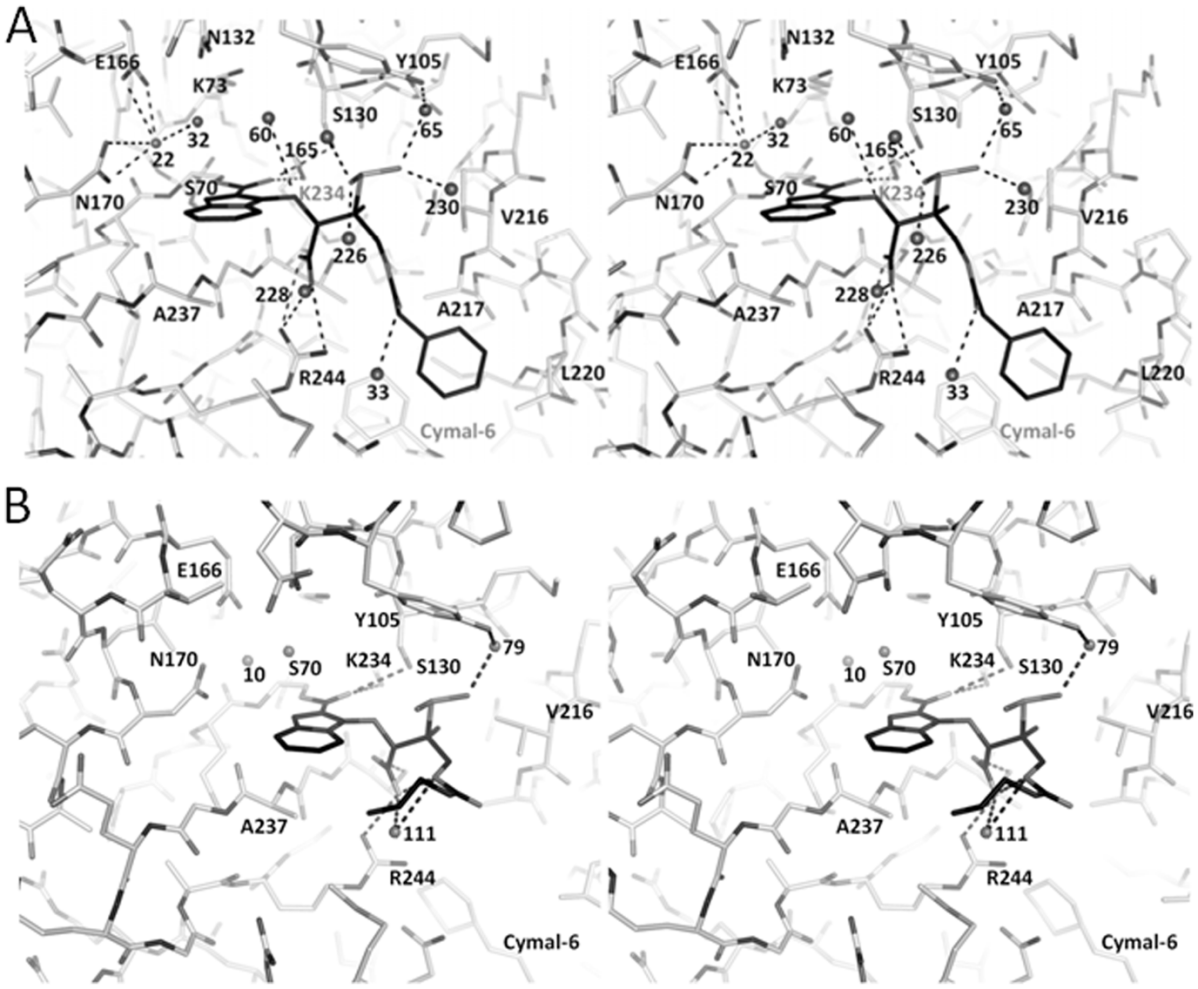
Penam sulfone interactions in SHV-1 active site. (A) Stereo view of SA1-204 bound to SHV-1. (B) Stereo view of SA3-53 bound to SHV-1.

### Data Collection and Structure Determination

X-ray diffraction data for the SHV-1: penem **1** complex was collected using the in-house Rigaku MicroMax-007 HF microfocus X-ray generator with Saturn 944+ CCD X-ray detector. Three hundred degrees of data for this SHV-1: penem **1** dataset was collected with 0.5° Δphi oscilliation steps at a single wavelength (Cu *K*α wavelength of 1.542 Å). The data were processed with both anomalous reflections kept separate and as well by merging them. The anomalous scaled dataset was subsequently used to generate an anomalous difference Fourier map using the *FFT* program [17] of *CCP*4 suite to identify the positions of sulfur atoms of the penem **1** intermediate.

**Figure 5:**
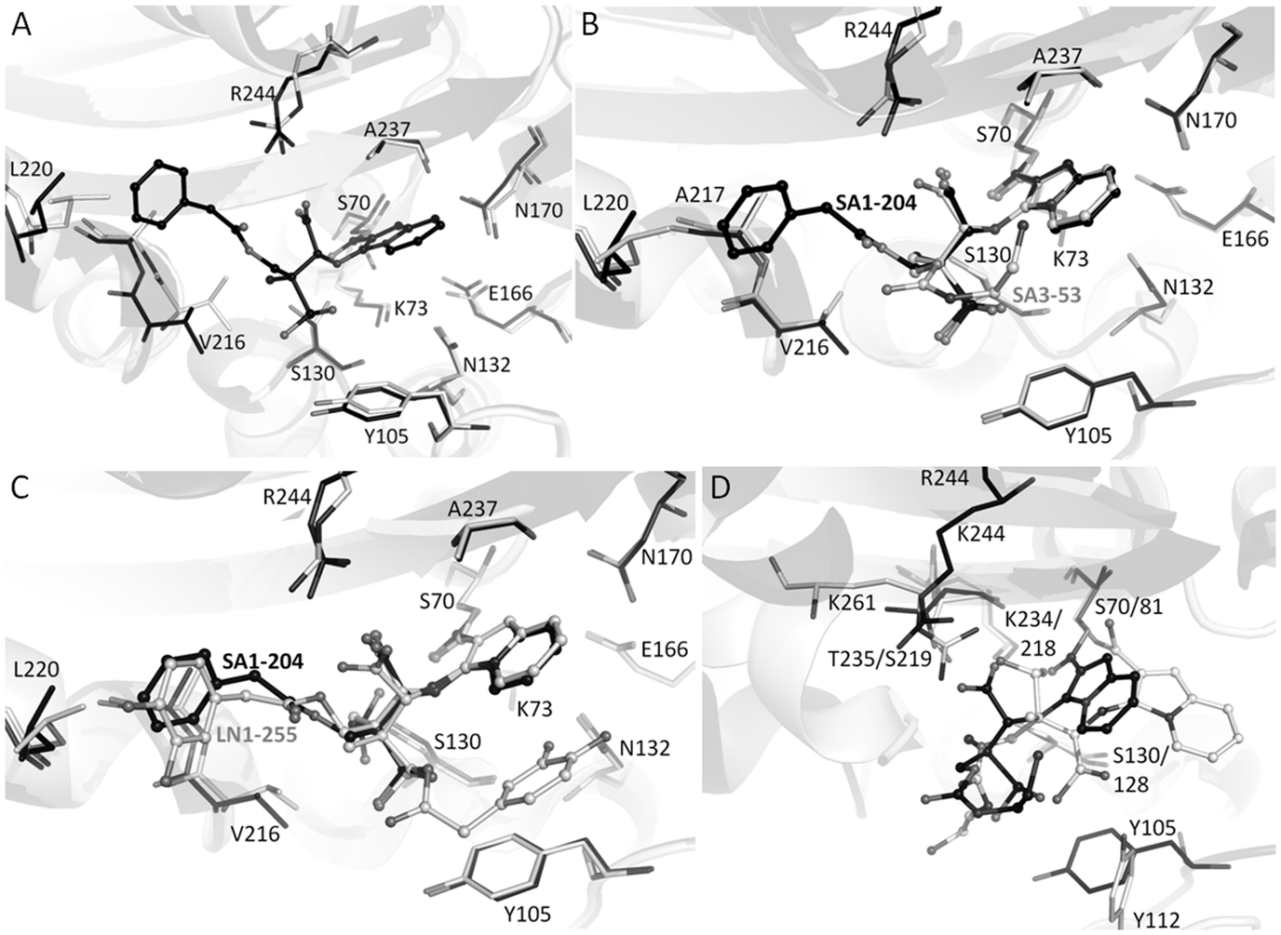
Structure superpositioning of penam sulfone complexes. (A) Superpositioning of SA1-204:SHV-1 (black) and apo *wt* SHV-1 (grey). (B) Superpositioning of SA1-204:SHV-1 (black) and SA3-53:SHV-1 (grey). (C) Superpositioning of SA1-204:SHV-1 (black) and LN1-255:SHV-1 (grey). (D) Superpositioning of SA3-53:SHV-1 (black) and SA3-53:OXA-24 (grey, PDBid 3FZC). The following atoms are used for superpositioning: SHV-1 residues 68–77, 232–237, 128–134, 243–245 were superpositioned onto OXA-24 residues 79–88, 216–221, 126–132, and 229–231, respectively. This yielded a r.m.s.d. 0.536 Å for 26Cα atoms.

X-ray diffraction data for the SHV-1:SA1-204 complex was collected at the Brookhaven National Synchrotron Light Source beamline X-29. Both the penem **1** and SA1-204 data sets were processed using HKL2000 [18].

**Table 2:** Minimum Inhibitory Concentrations (MICs) of Piperacillin (Pip) in Combination with 4 µg/ml Tazobactam (Tazo), SA1-204, and LN1-255.

	Piperacillin	Pip/Tazo (4 µg/ml)	Pip/SA1-204 (4 µg/ml)	Pip/LN1-255 (4 µg/ml)
	*µg/ml*	*µg/ml*	*µg/ml*	*µg/ml*
*E. coli* DH10B	2	1	1	4
*E. coli* SHV-1	2048	1024	64	32
*E. coli* SHV-2 (G238S)	256	2	8	4
*E. coli* SHV-5 (G238S/E240K)	1024	128	16	4
*E. coli* SHV-30 (R43SE240K)	128	1	2	4
*E. coli* R164H	1024	2	16	
*E. coli* R164S	32	1	2	
*E. coli* D179N	128	1	16	
*E. coli* SHV-49 (M69I)	256	128	32	32
*E. coli* S130G	128	64	64	64
*E. coli* R244S	64	8	32	32

**Table 3:** Minimum Inhibitory Concentrations (MICs) of Piperacillin (Pip) in Combination with 4 µg/ml Tazobactam (Tazo), and Penem 1.

	Piperacillin	Pip/Tazo (4 µg/ml)	Pip/Penem 1 (4 µg/ml)
	*µg/ml*	*µg/ml*	*µg/ml*
*E. coli* DH10B	4	2	2
*E. coli* SHV-1	2048	2048	8
*E. coli* SHV-2	256	4	4
*E. coli* R244S	64	16	4

Data for the SHV-1:SA-3-53 complex was collected at ALS beamline 4.2.2. and processed using D*trek [19]. Structure determinations were carried out using isomorphous replacement starting with chain A of the isomorphous crystal structure of SHV-1 β-lactamase (PDB 1VM1) [20]. Crystallographic refinement was carried out using REFMAC [21] and model building was done using COOT [22].

After initial refinement, density in the active site revealed a covalent intermediate attached to the S70 side chain. The PRODRG2 server [23] was used to obtain the parameter and topology files for the covalently attached inhibitor intermediates observed in the electron density maps (Figure 2). Crystallographic refinement was monitored using the program DDQ [24] and the final model quality was assessed using PROCHECK [25]. Data collection and refinement statistics are summarized in Table 1.

### Minimum Inhibitory Concentrations (MICs)

Chemical synthesis for the compounds used have been previously described for SA1-204 [4], SA3-53 [8], and penem **1** [3]. MICs were performed as reported earlier [5]. We compared the combination of piperacilin/SA1-204 to *i*) piperacillin/tazobactam (one of the most widely used β-lactam β-lactamse inhibitor combination in hospitals), *ii*) piperacillin/penem **1**, and *iii*) piperacillin/LN1-255 [5]. Each of these β-lactam β-lactamase inhibitor combinations was compared to piperacillin alone. MICs were also performed against *E. coli* DH10B strains that possessed variants of SHV-1 that contained amino acid substitutions which confer resistance to commercially available β-lactamase inhibitors and advanced generation cephalosporins. The strains were constructed as described.

### Kinetics

Kinetics for SA1-204 were performed by measuring *K*_m_ (*K*_i_) as a direct competition reaction as previously described [5].

## Results and Discussion

### SHV-1: Penem 1 Structure

The SHV-1: penem **1** structure was resolved at 1.84 Å. The initial unbiased *F*o-*F*c map of the penem **1** soaked SHV-1 crystal revealed electron density emanating from catalytic S70 residue (Figure 2A). Based on the shape of the density and the suspected reaction mechanism (Figure 1B), a 7-membered-ring acyl-intermediate was modeled. All 5 different possible species (intermediates **7–11,** Figure 1) were considered, yet after careful analysis of the stereochemistry and torsion angles, we concluded that penem **1** is in the *R*-isomer conformation (species **10**). This interpretation was aided by the measured torsion angles of 4°, defined by N4-C3-C-S, and 33°, defined by N4-C5-C6-C7. These two torsion angles are relative close to 0° indicating close to planarity due to the likely sp2 hybridization that we hypothesize to be present for these bonds representing species **10**. The dihedral angle around the bond involving atom C7 is 67° (defined by atoms C5-C6-C7-C). To further aid in the interpretation of the density, the anomalous signals of S atoms further confirmed positions of the two sulphur atoms of the modeled species **10** (Figure 2B). The acyl-enzyme intermediate for penem **1** is in agreement with a previous electrospray ionization mass spectroscopy study, although the precise enantiomer could not be identified from the mass alone [13]. In addition to penem **1**, the refinement also included a HEPES buffer molecule and one intact and one partial Cymal-6 molecule. 243 water molecules were examined carefully and added in the refinement including the deacylation water near residues E166 and N170. The final *R*/*R*_free_ is 18.7/23.4% and residues with phi-psi angles in the disallowed region of the Ramanchandran plot are not present (Table 1).

We collected a number of additional datasets with penem **1** at different soaking time points ranging from 7 minutes to 21 hours and all reveal the same single *R*-configuration at the C7 stereo center of the acyl intermediate (data not shown). The intermediate representing species **10** has a few direct hydrogen bonding interactions with the enzyme active site and has a number of additional van der Waals interactions (Figure 3A). The β-lactam carbonyl group is positioned in the oxyanion hole forming hydrogen-bonds with backbone nitrogen atoms of S70 and A237 (Figure 2A and 3A). In addition, the NH-thiazepine atom is hydrogen bonded to the carbonyl atom of S130 (3.2 Å) and the nitrogen of the bicyclic R1 side chain of penem **1** is hydrogen bonding to N132. As a result of forming the 7 membered heterocycle, the C3 carboxylate group points outwards and has 2.9–3.2 Å distance water-mediated interactions with D104 and S106. The heterocyclic substitution lies above the polar side chain of N170 and also points toward the bulk solvent. We emphasize that the C3 carboxylate of penem **1** is not in the vicinity of R244, a residue previously been shown to be important and affect the *K*_m_ (*K*_i_) of penem **1** about 100-fold, when substituted [13]. In the pre-acylation steps residue R244, either directly or indirectly, has a role in binding the carboxyl moiety of substrates and inhibitors in the active site of β-lactamases [26]. The discrepancy with our covalently bound structure suggests that the *K*_m_ (*K*_i_) value is more dependent on the initial interactions of the inhibitors than on interactions made by a later inhibitory intermediate such as observed crystallographically. Note that none of the previous β-lactamase:penem complexes have the penem carboxyl moiety interacting with R244 or with an equivalent arginine residue at that position (these structures will be discussed below). The likely reason for the carboxyl moiety not interacting with R244 in the crystallographically observed penem complexes is that once the inhibition pathway has reached the state of the 7-membered ring intermediate, the C6-C5 conjugated bond of this 7-membered ring is in resonance with the carbonyl bond thereby restricting torsion angle changes that might be needed to have the carboxyl moiety move towards R244. Also, the 7-membered ring is relatively bulky and rigid which also limits the reach of the carboxyl moiety even if the C6-C5 bond were not conjugated.

Regarding the protein structure conformation, the penem **1** bound protein structure is somewhat similar to that of the apo SHV-1 and SHV-1:penem **2** (PDB identifiers 1SHV and 1ONG, respectively) with r.m.s.d.s of 0.33 and 0.39 Å, respectively, of all Cα atoms in the superposition. Remarkably, the presence of the covalently bound penem **1** inhibitor induced several changes in the enzyme active site compared to both apo SHV-1 and SHV-1:penem **2** protein structures. The most prominent change is the outward shift of the loop containing Y105, which releases the steric hindrance between the C3 carboxylate group of penem **1** and the Y105 containing loop. This results in a 2.2 Å shift of the Cα position of Y105 compared to the apo SHV-1 and SHV-1:penem **2** structures (shown only for SHV-1:penem **2** in Figure 3B). A shift in the Y105 containing loop in SHV-1 was previously observed in SHV-1: boronic acid transition state inhibitor analogue bound structures [16](PDB identifiers 3MKF and 3MKE).

Compared with earlier crystallographic studies of similar penem inhibitors [10], [11](PDB identifiers 1ONG and 1ONH), the positions of the two ring systems of the covalently bound inhibitors vary greatly (Figure 3B–C). The orientation of penem **1** in the SHV-1 active site is more similar to that of the penem **2** in the GC1 active site than that of the previous SHV-1:penem **2** complex. Despite having unique orientations that differ from each other by a ∼180° rotation around the bond to the serine ester, all these three acyl-intermediates adopt the *R* configuration. This finding suggests that after acylation in SHV-1, the inhibitors undergo identical stereo specific chemistry, yet the final conformation is not the same; this is likely not a critical step in the inhibition process although the longevity of the cyclic inhibitory intermediate could depend on it. Notably, we observe that the deacylation water, held in place by residues E166 and N170, is present in the penem **1**:SHV-1 complex (Figure 3A). Displacement of this deacylation water is an additional chemical strategy that can improve the potency of the inhibitor by also slowing down deacylation.

Based upon these crystallographic findings (adoption of R conformer, deacylation water, carboxylate position) and previous observations, we conclude that the stability of the penem **1** intermediate is due to a different mechanism. We suggest that the decreased electrophilicity of the carbonyl carbon plays a major role; this decreaed electrophilicity is a result of the conjugation of the acyl ester with the large dihydrothiazipine ring [11]. The presence of the conjugation with the carbonyl bond is evidenced by the torsion angles of the O = C-C = C atoms (starting with the carbonyl oxygen) being all close to planar being 171, −10, and −23° for the penem **1**:SHV-1, penem **2**:SHV-1, and penem **3**:SHV-1 structures, respectively.

Interestingly, an earlier computational study predicted that penem **1** would form a dihydrothiazepine acyl-intermediate with the C7 *S* configuration [6] which is in disagreement with the crystallographically observed *R* configuration. Different conformations of the same penem inhibitor are not uncommon as a similar penem, penem **2**, also adopts different conformations in class A compared to class C β-lactamases [10]; or even within the same protein as for penem **3** [11](PDB identifier 1Q2Q).

Finally, we note that the penem **1** is also situated near a HEPES buffer molecule. HEPES was used in both this study’s crystallization protocol as well in the previous SHV-1 crystallization protocols to obtain the previous penem complexes [10]. The proximity of the sulfone moiety of HEPES could be used to design novel penem inhibitors with an added negatively charged substituent, similar to how the position of HEPES was used to rationally design the penam sulfone inhibitor SA2-13 [15].

### Penam Sulfone Structures

#### SHV-1:SA1-204 complex

The SHV-1:SA1-204 structure was determined at 1.53 Å resolution. The initial unbiased omit *F*o-*F*c map reveals a clear covalent acyl intermediate attached to the catalytic S70 residue with characteristic features including a bicyclic ring and a phenyl tail (Figure 2C). Based on the proposed reaction mechanism (Figure 1C), a bicyclic acyl intermediate was modeled, which fits well with the density and was included in refinement. In addition, 261 water molecules were added as well as one Cymal-6 and one fragment of Cymal-6 were included in refinement. The final *R*/*R*_free_ values were 16.8/19.4%; as above, residues were not in the disallowed region of the Ramanchandran plot (Table 1).

Based on previous Raman studies, the inhibition efficacy of SA1-204 was ascribed to its prolonged blocking of the SHV-1 enzyme active site as the unreacted Henri-Michaelis complex of up to one hour [12] although a follow-up Raman study suggested the inhibition does occur via reacting with S70 [27] as was observed in this study. In our study of the SHV-1: SA1-204 complex, the carbonyl oxygen of SA1-204 is positioned *out* of the oxyanion hole and stabilized by side chains of S130 and K234 (Figure 4A). The bicyclic ring partially occludes the oxyanion hole and makes van der Waals interactions with A237. Importantly, the C3-carboxylate group is noted to form a salt-bridge interaction with R244. Additionally, the sulfone group of SA1-204 interacts with water molecules including a water-mediated interaction with Y105 (Figure 4A). The phenyl tail of SA1-204 is in van der Waals distance with V216, A217, and L220 and is also close to a Cymal-6 molecule (Figure 4A). The C2-methyl group is likewise in van der Waals distance with V216.

The SHV-1:SA1-204 protein structure has a similar conformation as that of the structures of apo SHV and SHV-1:LN1-255 complex (PDB identifier 3D4F) with rmsd of 0.357 and 0.109 Å, respectively, with all Cα superpositioning. Compared with apo SHV-1 structure, the most prominent movements of the active site residues are the different rotamer taken by S130 and the outward shift of the V216 containing loop to accommodate the carbonyl oxygen and the C2-methyl group of SA1-204 intermediate (Figure 5A). The reorientation of the ester carbonyl away from the oxyanion hole and pointing toward S130 was previously observed and may contribute to the slow deacylation rate of imipenem inactivating TEM-1, meropenem inactivating SHV-1, and LN1-255 against SHV-1 β-lactamases although additional factors may play a role as well [5], [28]. Other potential reasons for the decreased deacylation rate and stability of penam sulfone intermediate include the steric and electrostatic barriers and the spatially increased distance for the approach of the deacylation water to the ester carbonyl (Figure 3B). Firstly, in this SHV-1:penam sulfone structure, the deacylation water is positioned 4.07 Å away from the ester carbonyl carbon compared while in the usual acyl intermediate this distance is 2.8 Å. Secondly, the bicyclic aromatic ring decreased the electrophilicity of the ester carbonyl due to the conjugating effect. Lastly, the bulky bicyclic aromatic ring imposes steric hindrance to the approach of the deacylation water to the ester carbonyl. SA1-204 is very similar to LN1-255 differing only by two hydroxyl moieties. Comparison of the inhibition data for these two penam sulfones indicates that SA1-204 is more potent than LN1-255 (considering IC50 values against three representative serine β-lactamases) [4]. The IC50 values are 0.001, 0.04 and 0.39 µM of SA1-204 against P99, TEM-1 and PC1 β-lactamase, respectively, whereas these values are 0.026, 0.06 and 0.7 µM, respectively, of LN1-255. The apparent improved *in vitro* affinity of SA1-204 could be due to the more hydrophobic nature of the C2 substituent: SA1-204 is an analogue of LN1-255 and contains a C2-phenylacetate substitution instead of a C2-catecholicacetate group (Figure 1). This C2 substitution of LN1-255 has two alternative conformations when complexed with SHV-1 whereas SA1-204 has only one conformation in the SHV-1 active site (Figure 5C); this is likely due to SA1-204’s phenyl ring seeking tighter hydrophobic interactions. However, LN1-255 possesses slightly better inhibitory properties compared to SA1-204 for Bla1 (class A) and Bla2 (class B) β-lactamases from *Bacillus anthracis* [7]. A noted advantage of LN1-255 is that it contains catechol features of the dihydrophenyl ring to potentially improve the entry into bacteria via the iron chelating uptake pathway [5].

#### SHV-1:SA3-53 complex

The SHV-1:SA3-53 structure was determined at 1.60 Å resolution. The structure of SA3-53 revealed a similar conformation of the inhibitor compared to SA1-204 except for the tail regions (Figures 2D, 4B, and 5B). The bicyclic ring, carboyxyl moiety, sulfone moiety all superimposed well. A striking difference between the two structures is that the phenyl ring of SA1-204 points in a different direction compared to the corresponding ethylenediamine tail of SA3-53. The ethylenediamine tail of SA3-53 is also not well resolved as evidenced by the electron density map (Figure 2D).

The structure of SA3-53 bound to the Class D β-lactamase OXA-24 was previously determined [8](PDB identifier 3FZC). The superposition of the SHV-1 and OXA-24 each bound with SA3-53 revealed that the inhibitor forms the same bicyclic intermediate (Figure 5D). However, the conformation in the active site of the inhibitory intermediate is quite different between the two structures. Firstly, the carbonyl oxygen of SA3-53 occupies the oxyanion hole in OXA-24, but not in SHV-1 (Figure 5D). Secondly, the positions of the carboxyl and sulfone moieties are completely different between the two structures. This indicates that the same inhibitor can form the same complex in different classes of β-lactamases, yet adopt very different conformations within the active site. Although SA1-204 has been tested against a variety of different classes of β-lactamases, SA3-53 has only been characterized against the carbapenemase OXA-24 and found to have a *K*_m_ of 4.1 µM [8].

### MICs and Kinetics: Proof of Concept

Representative MICs are summarized in Tables 2 and 3. Four µg/ml of SA1-204 added to piperacillin was as potent as an equal amount of tazobactam combined with piperacillin against a number of *E. coli* isolates containing *bla*_SHV_ (Table 2). In particular, SA1-204 and LN1-255 are quite potent against SHV-5 β-lactamase with LN1-255 being the most effective (Table 2). A possible explanation is that both SA1-204 and LN1-255 position their bicyclic aromatic ring system towards the direction of where the SHV-5 mutations are located (residues 238 and 240) and as such could provide favourable interactions in this region of the active site of SHV-5. Furthermore, LN1-255 adopts two conformations in the active site of SHV-1 (Figure 5C) with one conformation providing stacking interactions with the bicyclic aromatic ring system (Figure 5C); this latter interaction could thus potentially indirectly improve active site interactions with SHV-5 and as such provide a possible explanation for LN1-255 efficacy towards SHV-5. In contrast, SA1-204 and piperacillin also lowers MICs against *E. coli* bearing strains that contain substitutions in the Ω loop that confer the ESBL phenotype (R164S,-H and D179N), although not as effective as tazobacam and piperacillin. SA1-204 was slightly better than tazobactam against *E. coli* DH10B strains bearing the M69I substitution (IR phenotype). Against the *E. coli* DH10B strain that contained the S130G IR mutations, SA1-204 was equivalent to tazobactam when combined with piperacillin. Kinetic analysis revealed that *K*_m_ *(K_i_)* value of SA1-204 were in the nM range against SHV-1 (0.042±0.004 µM). This low *K*_m_ *(K_i_)* and demonstrated potency in cell based are observations that support the impact of the crystallographic structures.

In Table 3, we summarize our studies that compare 4 µg/ml penem **1** and piperacillin to equivalent amounts of tazobactam and piperacillin. Against strains harboring the wt SHV-1, the ESBL SHV-2 and the IR R244S, penem **1** was more potent than tazobactam when paired with piperacillin. The penem **1** structure reveals a well-ordered stable complex that likely contributes to the low MIC values of penem **1**.

In conclusion, we present the crystal structure of SHV-1 β-lactamase, the main β-lactam resistance determinant found in *Klebsiella pneumoniae,* bound with penem **1** and the two penam sulfones, SA1-204 and SA3-53. Despite the chemical similarity of these penicillin sulfones, we show that the final structure of the covalent adduct formed by each inhibitor can be very different. More importantly, these three structures reveal that conjugation of the carbonyl is a important mechanism that plays likely a key role in slowing deacylation. The detailed crystallographic insights gained from this study, especially in the context of increased resistance mediated by β-lactamases, could be used to further the design of new inhibitors.
